# Does Diversity Matter? Behavioural Differences between Piglets Given Diverse or Similar Forms of Enrichment Pre-Weaning

**DOI:** 10.3390/ani10101837

**Published:** 2020-10-09

**Authors:** Océane Schmitt, Aurélie Poidevin, Keelin O’Driscoll

**Affiliations:** 1 Pig Development Department, Teagasc Animal and Grassland Research and Innovation Centre, Moorepark, P61 C996 Fermoy, County Cork, Ireland; keelin.odriscoll@teagasc.ie; 2 Department of Animal Production, Easter Bush Veterinary Centre, Royal (Dick) School of Veterinary Studies, The University of Edinburgh, Easter Bush Campus, Midlothian EH25 9RG, UK; 3 Animal Behaviour and Welfare Team, Animal and Veterinary Sciences Research Group, SRUC, West Mains Road, Edinburgh EH9 3JG, UK; 4 Laboratoire d’Ethologie Expérimentale et Comparée EA 4443, Université Paris 13, Sorbonne Paris Cité, 93430 Villetaneuse, France; aureliepoidevin@gmail.com

**Keywords:** pigs, enrichment, welfare, biting, pre-weaning

## Abstract

**Simple Summary:**

This study looked at behavioural differences between piglets provided with either two identical or two different enrichment materials. From seven days-old until weaning, piglets were given hessian fabrics or bamboo: the control group had only hessian, and the diverse groups had both hessian and bamboo. One object was attached to the pen wall and the other suspended in the middle of the pen and piglets behaviours were observed. Objects suspended in the middle of the pen attracted more attention than objects attached to the wall. Piglets preferred hessian over bamboo, and the interest in hessian increased over time, while interest in bamboo remained unchanged. Piglets interacted differently with the objects: more oral manipulation and shaking occurred with the hessian and more pushing occurred with the bamboo. Control piglets performed more biting than piglets with diverse enrichment, both pre- and post-weaning. Therefore, providing different forms of enrichment to piglets pre-weaning has the potential to reduce biting occurrences and, thus, to promote welfare. Hessian was probably favoured because it allowed oral manipulation, which was the most frequent interaction observed.

**Abstract:**

This study investigated the behavioural effects of providing different enrichment materials to suckling piglets from 7 days-old until weaning. One object was attached to the pen wall (WALL), and the other was suspended in the middle of the pen (MID). Control group had the hessian fabric in both locations, and the two diverse groups had hessian and bamboo stick in alternate locations (i.e., BMID-HWALL and HMID-BWALL). Piglets behaviour was recorded on D0 (object introduction), D1, D5, D8, D12, and D14; at weaning and 1, 3, 5 and 15 days after. Groups did not differ in approaching or interacting with objects on D0. MID objects attracted more attention than WALL objects (*p* < 0.01). Piglets interacted more with hessian than bamboo (*p* < 0.001). They performed more oral manipulation and shaking with hessian (*p* < 0.001), but more pushing of bamboo (*p* < 0.001). Interactions with objects increased with time (*p* < 0.001), especially with hessian (*p* < 0.01), while interest in bamboo remained unchanged. Control piglets performed more biting than piglets with diverse enrichment (pooled data), both pre- and post-weaning (*p* < 0.05). Therefore, providing different types of enrichment material can reduce biting behaviour pre- and post-weaning. Hessian was favoured, possibly because this was easier to bite and shake, which were the behaviours most often observed.

## 1. Introduction

Provision of appropriate enrichment to pigs is mandatory in the European Union [1], to enable the performance of natural rooting and exploratory behaviour, and ultimately to reduce the occurrence of tail-biting and suppress the need for tail-docking [2]. Until recently, this legislation has not been fully enforced across the EU, and as such docking of tails is widespread; indeed, it is estimated that over 95% of pigs in the EU have their tails docked shortly after birth [3]. However, in recent years, the pressure to enforce from the European Commission (as well as the formation of the pig subgroup in the Animal Welfare Platform), the member states, the public and animal welfare organisations has resulted in renewed efforts by producers and researchers to identify management strategies, which will enable compliance and, in particular, methods of providing adequate environmental enrichment. 

Provision of enrichment can prevent or reduce tail-directed behaviours [4,5], improve pigs’ emotional state [6] and improve pig performance [7]. In general, loose organic material, such as straw (from a handful to deep straw-bedding), is considered to be the gold standard. However, in partially or fully slatted systems, which predominated in many countries, loose material may fall through the slats and cause disruption to or blockages in the slurry systems. A recent review of studies using non-straw enrichment found that the objects had to be exchanged regularly to significantly reduce tail biting [8]. They also highlighted the need to investigate further the behavioural needs addressed by these objects to promote their efficiency. For instance, destructibility and edibility are factors that extend the interest of the pigs in the objects [9].

Most published studies have investigated enrichment strategies after weaning, and as such, there is somewhat of a need for more research into how pre-weaning environmental enrichment can affect the occurrence of biting behaviours not only pre-weaning but after weaning as well. Telkänranta and colleagues [10] found that providing chewable enrichment (sisal rope and newspaper) reduced the occurrence of pen mate manipulation pre-weaning and the severity of tail damage post-weaning, relative to pigs who only had in addition to harder materials (wood shavings and a plastic ball attached to a chain). Brajon et al. [11] found that straw bedding increased animal comfort pre-weaning and reduced biting in the first six days post-weaning. However, they also theorised that the removal of enrichment post-weaning could lead to distress, as pigs who moved from a pre-weaning loose pen enriched with straw performed more belly-nosing than piglets who had been managed in a standard farrowing crate during lactation [11]. Finally, the study by Martin and colleagues [12] found that piglets from a pre-weaning enriched environment (i.e., increased space allowance and straw) played more pre-weaning than piglets in a barren environment and had better performance in an object recognition task and lower level of chronic aggression post-weaning. Therefore, the authors concluded that a complex-enriched environment might improve the development of the socio-cognitive skills of the piglets.

In reality, many farms have standard farrowing crates, which do not allow for the provision of loose materials, such as straw, nor can be easily adapted to provide more space. Moreover, producers consider the durability and longevity of a material when they are choosing what to provide, and items that can be washed, sterilised and reused over several batches could be favourable to them. Indeed, in a survey of producers carried out by Haigh and O’Driscoll [13], longevity was considered an important consideration for 97% of respondents. However, concern about the effectiveness of an enrichment material was also mentioned by 83% of respondents. Thus, our experiment aimed to compare two types of enrichment material for pre-weaned piglets, one being easily deformable and chewable (hessian fabric), yet which would need to be replaced for each batch, and the other being hard and non-chewable (bamboo stick), but which could be re-used from batch to batch. We hypothesised that the hessian would be more effective at engaging the piglets as its qualities are appealing to pigs [9]. However, we also hypothesised that provision of both types of material, rather than hessian alone, would reduce the performance of negative behaviours, both pre and post-weaning, and increase the performance of the play, as the provision of a more complex environment is considered to be positive for welfare [12]. 

## 2. Materials and Methods 

### 2.1. Ethical Statement

Ethical approval for this study was granted by the Teagasc Animal Ethics Committee (application TAEC133/2016). The experiment was carried out in accordance with the Irish legislation (SI no. 543/2012) and the EU Directive 2010/63/EU for animal experiments. At the end of the experiment, animals were returned to the commercial herd.

### 2.2. Animals and Management

This study used a total of 481 piglets from 36 litters, over three batches, and was performed at the Teagasc Moorepark pig research facility between March and May 2017. Each batch included 4 litters in each of the three treatment groups. The average litter size at the start of the experiment was 13.4 piglets (range: 8 to 15 piglets). 

Animals were managed as per normal farm practices: piglets were born into conventional farrowing pens (250 × 181 cm), which contained a sow crate (225 × 60 cm) and a heating pad (155 × 37 cm; 2/3 covered). Piglets’ teeth were clipped on the day of farrowing, tails were docked one-day post-farrowing, and males were not castrated. They received an injection of iron (Gleptosil^®^, Ceva, Amersham, UK) at four days post-farrowing and were vaccinated against Porcine Circovirus type 2 (Porcilis^®^ PCV ID, MSD, Kenilworth, NJ, USA) on the day of weaning (27 ± 0.1 days of age). At 10 days post farrowing, creep feed was introduced to habituate the piglets to post-weaning solid feeding. Details of the sow diets and creep feed given to piglets during lactation can be found in Schmitt et al. [14]. 

Piglets were assigned into groups of 12 animals at weaning, with litters within the same treatment group mixed together (pseudo-random mixing based on weight). Piglets were transported as litter groups by a wheelbarrow to the weaner accommodation and mixed with piglets from other litters in the weaner pen. Weaner pens were 2.4 m × 2.6 m in dimension with fully slatted plastic floors. The temperature was maintained at 27–28 °C immediately post-weaning by a computer-controlled heating and mechanical ventilation and reduced 2 degrees every 2 weeks thereafter in the weaner house. Artificial lighting (around 150 lux) was provided between 07:00 and 17:00 to supplement natural light from windows and promote a normal circadian rhythm.

Post-weaning, pelleted feed was provided ad libitum by a wet-dry feeding system consisting of single-space feeders. Water was also provided ad libitum in a separate nipple drinker. During the first week post-weaning, a starter diet was provided (Startrite 88, Provimi, Naas, Ireland), then a standard home-milled link diet for 2 weeks, before a standard commercial weaner diet was provided (87.6% dry matter, 18.5% protein, 6.7% fat, and had net energy of 10.3 MJ/kg). 

### 2.3. Enrichment

**Figure 1:**
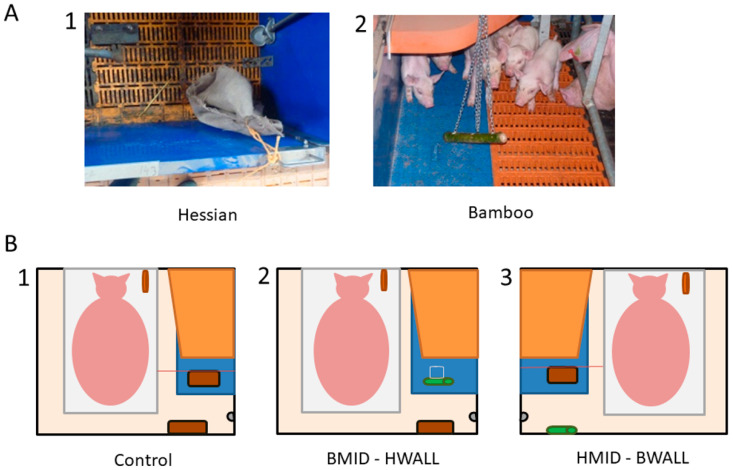
Objects used as piglet enrichment (**A**): Hessian fabric (1) and bamboo stick (2); schematic representation of the treatment groups (**B**): (1) control, two hessian fabrics, (2) diversity 1 group (BMID-HWALL: a bamboo stick in the middle (BMID) and a hessian fabric on the wall (HWALL)), (3) diversity 2 group (HMID-BWALL: a hessian fabric in the middle (HMID) and a bamboo stick on the wall (BWALL)). Each pen was equipped with a heating mat for piglets (blue area) covered by a canopy roof (orange area) and a piglet drinker fixed to the wall (in grey).

At approximately 1-week post farrowing (6.8 ± 0.8 days), two enrichment objects were provided in each pen, which only the piglets had access to. Objects were made of one of two materials: hessian fabric and a bamboo stick (Figure 1A). These were selected because they are a natural material, destructible and without danger for the animal (as per legal recommendation from EU Council Directive 2008/120/EC) and because they should not obstruct the slurry system. The bamboo stick was fastened to the pen using a chain, which was also accessible to the piglets. Although they do not meet the ‘natural and destructible’ criteria, chains are often used as forms of enrichment on pig farms [13]. The hessian fabric (20 × 20 cm) was attached to the pen using orange nylon ropes. The objects remained in situ until weaning. 

Objects were placed at one of two locations in the pen to assess and control for the effect of pen location on use of objects by piglets: (1) suspended in the middle of the pen (MID) and (2) suspended at the pen wall (WALL). These two locations were selected as they were the most accessible to piglets without risk of interference from the sow.

At weaning, each weaner pen was provided with a rubber floor toy (Easyfix^®^ LUNA 117; Easyfix, Ballinasloe, Ireland), which was the only enrichment given to all treatments, as per normal farm practices.

### 2.4. Treatment Groups

Enrichment materials were pseudo-randomly assigned to litters, making sure that the same enrichment was not assigned to two adjacent pens. All treatment groups received two items of enrichment. Control groups (CON) received two pieces of hessian fabric, while “diverse” groups received one piece of hessian and one bamboo stick. For ethical reasons, we did not include treatment with only the bamboo stick, as we considered that this enrichment did not sufficiently incorporate the characteristics of an appropriate enrichment material (e.g., it is not ‘destructible’). Within the ‘diverse’ groups, half of the groups received the bamboo stick in the middle of the pen and the hessian fabric at the pen wall adjacent to the room corridor (BMID-HWALL), and the other half of the groups received the materials the other way around (HMID-BWALL) (Figure 1B). In all groups, a rope was attached to the sow pen, as a form of enrichment for her. Piglets could reach this rope as they grew larger. 

### 2.5. Measurements

#### 2.5.1. Behavioural Observations

Reaction to novelty. Immediately following the introduction of the enrichment in the pens (D0), piglets were video recorded for 5 min (camera: Panasonic HC-250EB-K, Panasonic^®^, Currys PC world, Carlow, Ireland; fixed on a tripod). The latency for the first piglet to touch either of the objects was recorded. In addition, the number of interactions with both enrichment objects was recorded at the pen level. Videos were analysed using The Observer XT software (Noldus, Wageningen, The Netherlands) using all occurrence continuous sampling. 

Routine observations. Prior to weaning, direct behavioural observations were carried out from the side of the pen on D1, D5, D8, D12 and D14 of the experiment. Post-weaning, observations were carried out on D1, D3, D5 and D15 after the day of weaning. Data were collected on a recording sheet using all occurrence continuous sampling, at pen level. 

For all observation days, seven 3-minutes observation sessions were conducted for each pen, one each hour between 09:00 and 16:00. The order of observation was randomised for each recording day, and all occurrences of all piglets behaviours were recorded. Both general behaviours, which could be carried out in all locations in pen, and specific object-orientated behaviours were recorded, as described in the ethogram in Table 1. All negative behaviours (biting, belly-nosing and aggression) were also consolidated into a single measure of negative behaviour. The same ethogram was used, both pre- and post-weaning, other than suckling and udder massage, which were only possible pre-weaning.

#### 2.5.2. Body Lesions at Weaning

At weaning, each piglet was scored for presence (score 1) or absence (score 0) of lesions (i.e., scratches) on the body, snout, tail and ears. 

### 2.6. Statistical Analysis

Statistical analyses were performed using SAS 9.4 (SAS Inst. Inc., Cary, CA, USA). The experimental unit for the analysis was the litter. General linear models (GLM) and generalized linear mixed models (GLMM) were fitted using the Residual Pseudo Likelihood approximation method. Differences were considered statistically significant when alpha was ≤0.05, and tendencies were determined when alpha was between 0.05 and 0.1. 

For all measures, an overall treatment effect was initially investigated, as well as a comparison between the control and diversity treatments considered together. The separate analysis then investigated differences in interactions with the enrichment materials, depending on the type provided and its location in pen. As the experiment did not include a full cross-over design, we were unable to investigate the interaction between the two. As such, we investigated the difference between each material and location combination across all three treatments.

#### 2.6.1. Reaction to Novelty

The latency for piglets to interact with the enrichment items once they were placed in the pen was compared using the Kruskal–Wallis test. In several pens, there was no interaction within 3 min. As such, data were also compared using survival analysis (Proc Lifetest) so that pens, where no interaction took place, could be censored from the data. 

The number of interactions with the enrichment materials within the first five minutes of introduction was analysed using a generalised linear mixed model (Proc Glimmix). A Poisson distribution was specified, as the data consisted of counts of the number of interactions. For the overall group effect, fixed effects included treatment group (*n* = 3) and replicate (*n* = 3), with piglet number included as a continuous covariate. The difference between the control and diversity groups was also included using a contrast statement. Sow and location of enrichment within the pen were also included as random effects.

For the second analysis, fixed effects were enrichment location in pen, the material used, and both the material and location nested within the treatment group. Random effects and covariates were as before.

#### 2.6.2. Interaction with Enrichment

Prior to analysis, the data were averaged across the 7 observation periods per day. A general linear model (Proc Mixed) was used to investigate the interaction with enrichment across the three treatment groups. Data were averaged for each recording day. Treatment, day and their interaction and replicate were fixed effects in the models. The number of piglets in the litter was included as a covariate, and the day was considered a repeated effect. The sow was considered a random effect. 

For the second analysis, the total of all behaviours, as well as the instances of oral, shaking and nosing behaviours, was investigated using another general linear model. Fixed effects were the location in pen, the material used, both the material and location nested within the treatment group, day and relevant interactions. Both day and the location of the enrichment were considered repeated effects, and a compound symmetry “unstructured covariance structure” specified. Random effects and covariates were as before. The slice function was used to investigate differences over time within each material/location combination. 

#### 2.6.3. General Behaviour

Linear mixed models were used to analyse the following behaviours prior to weaning: the total number of piglets in the pen suckling, at the udder, asleep, playing individually, playing socially, total playing and biting other piglets. Aggression was only seen twice prior to weaning, and belly nosing of other piglets was not seen and so was not analysed. Fixed effects included treatment group and time and their interaction, as well as replicate. The number of piglets in pen was included as a covariate for the analysis of total behaviours. The repeated effect of the day was accounted for, using either an autoregressive or autoregressive heterogeneous covariance structure, depending on the best fit. The sow was included as a random effect.

Post-weaning, the following behaviours were analysed using mixed models as before: numbers asleep, playing alone, playing socially, total playing, biting and a total of negative behaviours (biting, aggression and belly nosing) and interaction with the enrichment. The amount of aggression and belly-nosing on each recording day was analysed using Kruskal–Wallis tests. Multiple comparisons were taken into account using a post hoc Bonferroni test.

## 3. Results

### 3.1. Animal Behaviour

#### 3.1.1. Reaction to Novelty

There were no differences between the three treatment groups in terms of latency to approach the objects on the first day of the experiment. On average, control piglets took 195.3 ± 38.54 s to approach, BMID-HWALL piglets took 127.6 ± 38.54 s to approach, and HMID-BWALL piglets took 148.5 ± 38.54 s to approach (F_2, 33_ = 0.81, *p* = 0.45).

There was no effect of treatment group or difference between the control and diverse groups when it came to the number of interactions that piglets had with the objects during the first five minutes after introduction. Neither was there an effect of the type of object. However, there was a strong effect of location in pen (*p* < 0.001), with items in the middle of the pen receiving more attention than those at the wall (1 (0–14), vs. 0 (0–1), respectively; median (interquartile range)). 

With regard to differences between the materials depending on their location and treatment group, we found that there tended to be an overall effect of material and location combination (*p* = 0.1). There were more interactions with the object at the MID location compared to the object at the WALL location. Indeed, when in the same pen, there were more interactions with bamboo at MID compared to hessian at WALL (5 (0–21.5) vs. 0 (0–3.5), respectively; *p* < 0.01), and there were more interactions with hessian at MID compared to bamboo at WALL (2.5 (0–13) vs. 0 (0–0), respectively; *p* < 0.01). There were also more interactions with bamboo when it was at MID rather than WALL (5 (0–21.5) vs. 0 (0–0), respectively; *p* = 0.05).

#### 3.1.2. Interaction with Enrichment

**Figure 2:**
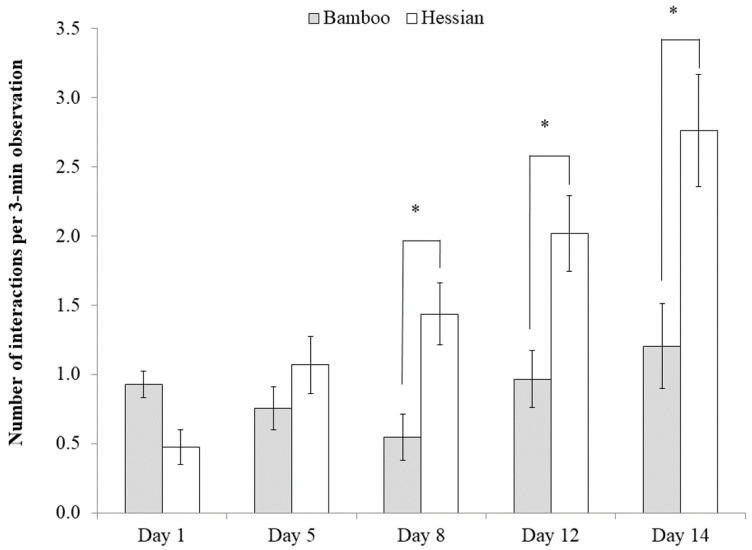
The number of interactions with different materials on each experimental day pre-weaning. There was an interactive effect between the material and experimental day (*p* < 0.001). * indicates a significant difference between the objects at *p* < 0.05.

The number of interactions with objects was dependent on their location in the pen; piglets interacted more with material suspended in the middle of the pen than with material hanging on the wall (1.41 ± 0.10 vs. 1.02 ± 0.11, respectively; t_43.2_ = 3.55, *p* < 0.001). Piglets also interacted more overall with hessian than bamboo (1.55 ± 0.10 vs. 0.88 ± 0.13, respectively; t_62.6_ = −4.48, *p* < 0.001). Overall, piglets’ interactions with the objects increased over time (F_4, 55.9_ = 6.75, *p* < 0.001), and there was an interaction between day and material on the number of interactions performed (F_4, 60_ = 10.14, *p* < 0.001 (Figure 2). Indeed, piglets interacted more with the hessian than the bamboo on days 8 (t_65_ = −3.46, *p* < 0.05), 12 (t_62.1_ = −3.37, *p* < 0.05) and 14 (t_65.1_ = −3.35, *p* < 0.05).

**Figure 3:**
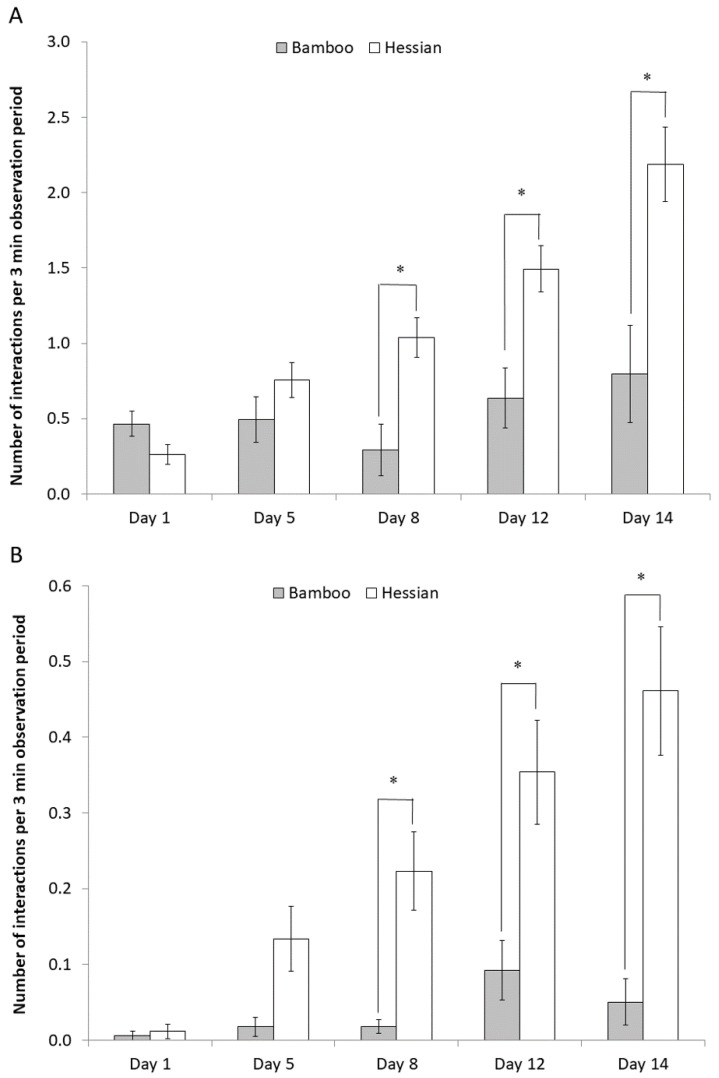
The number of oral manipulations (**A**) and shaking (**B**) behaviours on the different materials on different days pre-weaning. There was an overall effect of the interaction object * day for both behaviours (oral manipulations: F_4, 57.9_ = 9.91, *p* < 0.001; shaking: F_4, 62.9_ = 4.85, *p* < 0.005), and * indicates pairwise significant differences within a day at *p* < 0.05.

Oral behaviour was by far the most frequent type of behaviour observed. Overall, piglets performed approximately twice as much oral behaviour with hessian (1.15 ± 0.08 instances/3 min) than with the bamboo (0.54 ± 0.10 instances/3 min; t_56.6_ = −5.39, *p* < 0.001). There was also an interaction between day and material (Figure 3A; F_4, 57.9_ = 9.91, *p* < 0.001), with more of oral behaviour being directed towards hessian than bamboo on days 8 (t_64.7_ = −3.87, *p* < 0.01), 12 (t_58.2_ = −3.83, *p* = 0.01) and 14 (t_63.3_ = −3.85, *p* = 0.01) of the experiment. Likewise, pigs shook the hessian more than the bamboo on days 8 (t_67.3_ = −2.83; *p* < 0.05), 12 (t_65.8_ = −2.61; *p* = 0.05) and 14 (t_65.8_ = −3.4; *p* < 0.01) (Figure 3B). There was no difference between materials in the amount of nosing carried out (F_1, 45.7_ = 2.44, *p* > 0.1). Pushing of the material with the snout was primarily only directed towards bamboo; it was only observed being directed towards hessian at a very low level on Day 5 of the experiment (*p* < 0.001 for all days). Lying and mounting were infrequently observed, therefore limiting the statistical analysis, and (from the raw data) there was no difference in their frequency between the materials on any day.

**Figure 4:**
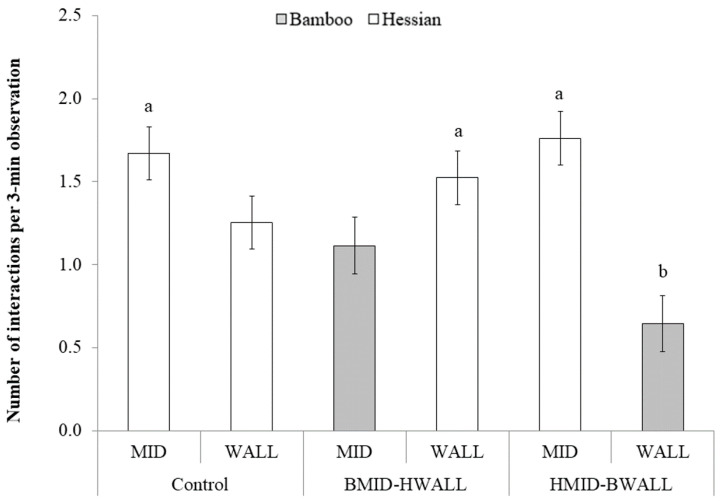
The number of interactions with different materials in different locations. The control group had two hessian fabrics; BMID-HWALL group had bamboo in the middle and a hessian fabric on the wall; HMID-BWALL group had a hessian fabric in the middle and bamboo on the wall. There was no overall effect of the interaction treatment * object * location (F_3, 44.1_ = 0.62, *p* = 0.6), but different letters (a, b) indicate pairwise significant differences at *p* < 0.05.

When it came to differences between the materials relative to where they were positioned in each group, there was no overall effect (treatment group * object * location: F_3, 54.2_ = 1.04, *p* = 0.38; Figure 4). However, piglets interacted less with the bamboo when it was located at WALL than with hessian at MID, whether the hessian was in the same pen (HMID-BWALL; t_45.6_ = −4.74; *p* < 0.001) or in another pen (Control; t_60.8_ = −3.44; *p* = 0.01). There was no significant difference in interaction when bamboo was located at MID and hessian was located at WALL in the same pen (BMID-HWALL; t_45.6_ = −2.37; *p* = 0.19). When considering only pens that had both bamboo and hessian available, bamboo at MID tended to attract less attention than hessian at MID (t_60_ = −2.77; *p* = 0.08).

**Figure 5:**
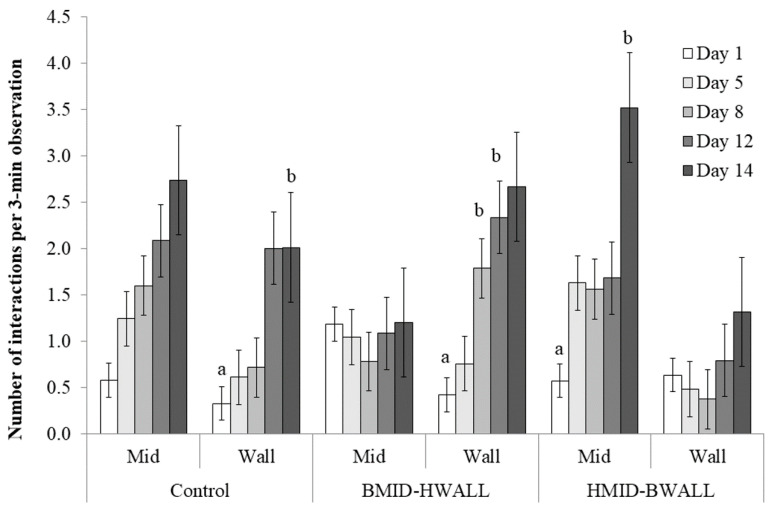
The evolution of interaction with enrichment over time, depending on the material provided and location. The control group had two hessian fabrics, BMID-HWALL had bamboo in the middle and hessian fabric next to the wall, and HMID-BWALL had the hessian fabric in the middle and bamboo on next to the wall. Different letters (a, b) indicate differences within material/location combination between days, at *p* < 0.05.

There was also an interaction between observation day and the object * location combination (treatment group * day * location * object: F_20, 119_ = 2.71, *p* < 0.001; Figure 5). There was no effect of day on the amount of interaction with bamboo, regardless of whether it was at WALL (F_4, 62.1_ = 0.35; *p* = 0.85) or MID (F_4, 61.8_ = 0.59; *p* = 0.67). However, for hessian, there was an effect of day regardless of the pen or location. In the control group, there was an overall effect of day on the number of interactions with hessian when located at MID (F_4, 61.7_ = 5.48; *p* < 0.001), with a tendency for more interactions on Day 12 than Day 1 (t_65.7_ = −3.66; *p* = 0.09), and when located at WALL (F_4, 61.7_ = 4.58; *p* < 0.005), with significantly more interactions on Day 12 than Day 1 (t_65.7_ = −4.07; *p* < 0.05). The overall effect of day on the number of interactions with hessian was also significant for BMID-HWALL (F_4, 62.1_ = 8.44, *p* < 0.001) and HMID-BWALL (F_4, 61.8_ = 6.84, *p* < 0.001) groups. Specifically, within BMID-HWALL, more piglets interacted with hessian on Day 8 (t_66.3_ = −3.96; *p* < 0.05) and Day 12 (t_66.5_ = −4.64; *p* < 0.01) than Day 1, and within HMID-BWALL, more pigs interacted with hessian on Day 14 than Day 1 (t_64.3_ = −4.57, *p* < 0.01).

Post-weaning, there was no effect of treatment group (F_2, 35_ = 1.42, *p* = 0.26), or interaction between group or day (F_6, 44.8_ = 0.7, *p* = 0.65), on the amount of interaction with the floor toy observed.

#### 3.1.3. General Behaviour

There was no overall effect of treatment group on biting behaviour prior to weaning, but pigs in the two diversity treatments (combined) performed less biting than those in control (*p* < 0.05; Table 2). Post-weaning, there was both an overall effect of treatment (*p* < 0.05), as well as a difference between the diversity treatments and control (*p* = 0.01). Indeed, control piglets performed more biting than BMID-HWALL piglets (5.2 ± 0.52 vs. 3.1 ± 0.55, respectively; t_26.2_ = 2.8, *p* < 0.05) (Table 2). All other behaviours did not differ between the treatments either pre- or post-weaning, including the total number of interactions with the enrichment objects (Table 2).

### 3.2. Body Lesions

Lesions on piglets’ body, snout, tail and ears were rare at weaning, and no difference in their prevalence was found between the different groups (Table 3).

## 4. Discussion

This study aimed to investigate the effectiveness of two types of pre-weaning enrichment material at engaging piglets, and in the main, the results confirmed our hypotheses. As expected, piglets engaged more with the more manipulable material (hessian), than with the harder substrate (bamboo). They predominately performed different types of behaviour when engaging with each material, likely related to the ease with which they could grab the material in their jaws. Moreover, we found that biting behaviour started to occur during the pre-weaning period and that the presence of two different types of enrichment in the farrowing pen, rather than two items of the same material, reduced it both pre- and post-weaning. However, this diversity of enrichment had no additional positive effect on the performance of play behaviour.

In contrast to the idea that piglets might lose interest over time, they instead increased the number of interactions with the enrichment objects. They showed a clear and growing interest in the material that was easy to manipulate and destructible, i.e., the hessian, rather than the object that was more resistant, i.e., the bamboo; as interactions with hessian increased in all treatment combinations, while interactions with bamboo remained at the same level across lactation. It is important to emphasize here that the objects were not renewed during the experiment, and thus the increase in interest was not due to the renewal of the hessian fabric. Therefore, our results suggest that the qualities of material used as enrichment influences whether interest in it is at least sustained and whether it will increase. An enrichment material that sustains interest is optimal for the producer, as it removes the need to renew or replace enrichment materials with novel items, thereby decreasing the staff workload in relation to providing enrichment. One concern was that the hessian might not have lasted for the duration of the lactation, but in all cases, hessian remained by the end of the study period. 

The increased engagement over time was likely due to the developmental stage of the piglets. Previous studies have also reported an increased level of engagement with enrichment as piglets age prior to weaning [17], and the fact that the increase was directed primarily towards the hessian fabric rather than the bamboo stick implies that this material was more appealing as time went by. This was likely due to the fact that it was chewable and could be grabbed in the mouth, which was not as easily possible with the bamboo stick, and evidenced by the greater performance of oral manipulatory behaviour and shaking, which could only easily be performed if the material could be gripped with the jaws. In contrast, more pushing with the snout was performed towards the bamboo; this could either be interpreted as thwarted attempts to bite or alternatively could simply be a different aspect of the normal behavioural repertoire. The nasal disk is used extensively in rooting behaviour and was used to push and move items while pig forage in a natural environment. Thus, the ability to perform pushing behaviour might have been rewarding in its own right. The composition of the hessian likely made it less responsive to a push, and the texture might have stimulated the piglets to grasp it in their mouths. Consequently, the provision of both materials might have allowed the piglets to perform a greater range of behaviours and satisfied more of their motivation to root.

The satisfaction of the rooting needs could explain the fact that the occurrence of biting behaviour pre- and post-weaning was lower in the diverse groups than the control. At face value, this could be considered surprising; as the hessian attracted more attention in general than the bamboo, theoretically providing two pieces of hessian in pen rather than one would attract more attention from the pigs, and divert them away from biting each other. However, as stated above, it is possible that the two different types of enrichment actually allowed piglets to perform a greater range of behaviours, and thus finding it easier to satisfy the behavioural need to explore and root. The ability to perform a greater range of behaviours associated with a particular activity allows an animal to satisfy their motivation to engage in that activity more quickly. For instance, in poultry, fewer ducks were observed in contact with a bath than a narrow chicken drinker, even though having access to open water is important to ducks [18]. The authors hypothesised that this was because, at the bath, ducks were able to perform their full complement of bathing behaviours, as well as drinking. At the narrow resource, only drinking was possible, so the ducks were observed more often in failed attempts to complete the action of head dipping prior to preening their feathers. These authors hypothesised that the importance of a resource to an animal, or the effectiveness of the resource in fulfilling the animal’s need, might be best indicated by the behaviours the animal performs and the frequency and duration of these behaviours while engaging with the resource, rather than simply time spent at the resource or numbers engaging with it [18]. Future research investigating enrichment materials should consider the range of behaviours, which the material enables the animals to perform, as well as considering the time spent interacting with it. Considering, in more detail, the components of foraging behaviour will allow a better comparison of materials and aid identification of the needs that are accommodated by the different objects or material [8]. 

Although there were differences in biting behaviour both before and after weaning, this was not reflected in lower skin lesion scores. This is likely because biting may not be very damaging during the pre-weaning period, as the piglets may not have the ability to bite through the skin due to their small size. In fact, it has previously been reported in older pigs that differences in performance of damaging behaviours are not always correlated with differences in the severity of lesions on the tails or ears [19,20]. It is also likely that the magnitude of the difference in biting behaviour is not great enough to have a biological effect when it comes to the ultimate outcome of damage to the skin. Nevertheless, given that the difference in the amount of damaging behaviour observed appeared to diverge even more after weaning than before, it is possible that the beneficial effects of the diverse enrichment may last into the weaner stage at least and reduce the risk of damage occurring later on. Further work could follow pigs longer into the weaner and finisher stages. The fact that the pigs’ tails were docked might also have reduced the likelihood that evidence of damaging behaviour would be present; docking the tail is the most effective method of reducing the risk of tail biting and the damage that ensues. It would be interesting to repeat such a study as this one, to determine whether the same benefits of providing piglets with more diverse enrichment in the farrowing pens exist when tails are not docked. 

We hypothesised that piglets might prefer the object placed in the middle area of the pen, as it was more accessible to them and allowed interactions from all sides. Indeed, when enrichment was initially introduced to the pens, piglets interacted more with the objects suspended in the middle than with the objects attached to the wall. Overall, this preference was maintained throughout the pre-weaning period, and when the same item was provided in both locations (hessian), more interactions were observed at the material in the middle of the pen. However, in diverse groups, the attractiveness of the hessian overrode the preference for using the material at the middle location; the only treatment where more observations were observed at the wall, albeit numerically, was the one with hessian located there and the bamboo in the middle of the pen. Therefore, it seems that the preference of piglets for an object is dependent on both type of material and its location in pen; both aspects should be considered when implementing enrichment on commercial farms and in experiments comparing the effectiveness of enrichment type.

## 5. Conclusions

Provision of two different enrichment materials, with differing qualities, in the farrowing pen could help to reduce the occurrence of biting behaviour in pigs during both the lactation and post-weaning stages. Piglets engaged more with the chewable and manipulable material, but at the same time, performed less biting behaviour when they were provided with both this type and a harder type of object. The hessian fabric did not need to be changed or renewed during the lactation period, despite being increasingly used by the piglets, and as such could be easy to implement on commercial farms, to supplement harder, more durable and long-lasting materials.

## Figures and Tables

**Table 1:** Ethogram used for the routine behavioural observation of the piglets during lactation and post-weaning periods.

	Behaviour	Definition
General Behaviour	Suckling	Teat in the mouth. Vigorous and rhythmic suckling movements. This behaviour begins when the sow grunts [15].
	Udder massage	Nose in contact with the udder, leaning against it. Ample and rhythmic up and down head movements [15].
	Sleeping	Lying down, eyes closed [15].
	Playing	Head shaking, springing (sudden jumping or leaping), running with vertical and horizontal bouncy movements. Can involve partners (chasing, jumping on each other...) [15].
	Play fight	The outcomes of social play interactions were recorded as play ending in aggression (forceful fighting with head knocking and biting the neck/shoulders and/or ears of another piglet) [16].
	Aggression	Focal piglet strikes or bites another piglet or attempts to do so (e.g., head/shoulder knocks). Forceful pushing with the head or body [15].
	Biting	Chewing or biting the tail, ear, snout of another pig [15].
	Belly-nosing	Repeated up and down massage movements with the snout onto another piglet or the sow (except the udder) [15].
Object Directed Behaviour	Oral manipulation	The piglet catches with its jaw and tightens it strongly or bites slightly and repeatedly the object.
	Shake	While holding an item in its mouth, the focal animal energetically moves the item from side to side using its neck and head [12].
	Push	The piglet uses its snout to move an item.
	Lying	The animal is lying on the object.
	Mounting	The piglet uses its legs to mount on another piglet or the object.
	Nosing	The snout is close to or in contact with a substrate [15].

**Table 2:** Mean ± S.E. number of behaviours performed during the 3-min observations pre-weaning (D07, D11, D14, D18 and D20) and post-weaning (D28, D32, D34, D40). The enrichment given to piglets was: two pieces of hessian fabrics (one suspended in the middle of the pen and one attached to the pen wall) for the control group; a bamboo stick suspended in the middle of the pen and a hessian bag attached to the pen wall for the BMID-HWALL group; a hessian bag suspended in the middle of the pen and a bamboo stick attached to the pen wall for the HMID-BWALL group. Different superscript letters (a, b) indicate significant differences between the treatment groups (*p* < 0.05).

	Control Treatment	Diversity Treatments		Treatment Effect (all)		Treatment Effect (Control vs. Diversity)	
		BMID-HWALL	HMID-BWALL	*F*-Value	*p*-Value	*F*-Value	*p*-Value
Pre-Weaning							
Bite	0.9 ± 0.09	0.6 ± 0.09	0.6 ± 0.09	F_2, 44.8_ = 2.04	0.14	F_1, 45.3_ = 4.06	0.05
Play	2.6 ± 0.29	2.6 ± 0.28	2.7 ± 0.29	F_2, 28.4_ = 0.11	0.90	F_1, 28.4_ = 0.02	0.88
Play-fight	0.9 ± 0.09	0.7 ± 0.09	0.7 ± 0.09	F_2, 30.5_ = 1.39	0.26	F_1, 30.6_ = 2.47	0.13
Suckling	2.4 ± 0.34	2.6 ± 0.34	2.5 ± 0.35	F_2, 29.8_ = 0.06	0.95	F_1, 29.9_ = 0.07	0.79
Udder massage	3.3 ± 0.31	3.0 ± 0.30	3.4 ± 0.31	F_2, 29.2_ = 0.52	0.60	F_1, 29.3_ = 0.2	0.65
Sleep	8.4 ± 0.27	8.1 ± 0.27	8.5 ± 0.27	F_2, 30.6_ = 0.55	0.58	F_1, 30.8_ = 0.16	0.69
Object interactions	1.25 ± 0.13	1.34 ± 0.12	1.12 ± 0.13	F_2, 51.1_ = 0.85	0.43	F_1, 51_ = 0.01	0.92
Post-Weaning							
Bite	5.2 ± 0.52 ^a^	3.1 ± 0.55 ^b^	3.8 ± 0.55	F_2, 26.2_ = 4.06	0.03	F_1, 26.2_ = 7.22	0.01
Play	4.8 ± 0.62	5.7 ± 0.64	4.6 ± 0.63	F_2, 28_ = 1.63	0.21	F_1, 28_ = 0.49	0.49
Play-fight	1.5 ± 0.23	1.5 ± 0.24	1.3 ± 0.24	F_2, 32.8_ = 0.34	0.71	F_1, 32.9_ = 0.22	0.64
Sleep	4.4 ± 0.32	5.0 ± 0.34	4.9 ± 0.33	F_2, 25.2_ = 0.87	0.43	F_1, 25.3_ = 1.65	0.21
Object interactions	8.6 ± 0.78	8.9 ± 0.82	7.1 ± 0.81	F_2, 35_ = 1.42	0.26	F_1, 35.3_ = 0.38	0.54

**Table 3:** Mean ± S.E. percentage of piglets with lesions on the body, snout, tail and ears at weaning.

	Control	Diversity 1 BMID-HWALL	Diversity 2 HMID-BWALL	*F*-Value	*p*-Value
Body lesions	23.4 ± 4.97	19.7 ± 5.05	20.1 ± 5.07	F_2, 33.3_ = 0.17	0.84
Snout lesions	9.9 ± 4.83	12.5 ± 4.88	13.7 ± 4.89	F_2, 33.2_ = 0.16	0.85
Tail lesions	9.1 ± 4.38	11.6 ± 4.43	17.0 ± 4.45	F_2, 33.3_ = 0.85	0.44
Ears lesions	60.6 ± 6.31	54.2 ± 6.40	55.2 ± 6.43	F_2, 33.1_ = 0.50	0.75
